# Unraveling the potential of endothelial progenitor cells as a treatment following ischemic stroke

**DOI:** 10.3389/fneur.2022.940682

**Published:** 2022-09-08

**Authors:** Antía Custodia, Alberto Ouro, João Sargento-Freitas, Marta Aramburu-Núñez, Juan Manuel Pías-Peleteiro, Pablo Hervella, Anna Rosell, Lino Ferreira, José Castillo, Daniel Romaus-Sanjurjo, Tomás Sobrino

**Affiliations:** ^1^NeuroAging Laboratory (NEURAL), Clinical Neurosciences Research Laboratory (LINC), Health Research Institute of Santiago de Compostela (IDIS), Santiago de Compostela, Spain; ^2^Centro Hospitalar e Universitário de Coimbra, Coimbra, Portugal; ^3^Faculdade de Medicina da Universidade de Coimbra, Coimbra, Portugal; ^4^Centro Neurociências e Biologia Celular, Coimbra, Portugal; ^5^Neuroimaging and Biotechnology Laboratory (NOBEL), Clinical Neurosciences Research Laboratory (LINC), Health Research Institute of Santiago de Compostela (IDIS), Santiago de Compostela, Spain; ^6^Neurovascular Research Laboratory, Vall d'Hebron Research Institute, Universitat Autònoma de Barcelona, Barcelona, Spain; ^7^CNC-Center for Neuroscience and Cell Biology, CIBB-Centre for Innovative Biomedicine and Biotechnology, UC, Biotech Parque Tecnológico de Cantanhede, University of Coimbra, Coimbra, Portugal

**Keywords:** angiogenesis, cell therapy, endothelial progenitor cells, secretome, stroke, vasculogenesis, cerebral ischemia

## Abstract

Ischemic stroke is becoming one of the most common causes of death and disability in developed countries. Since current therapeutic options are quite limited, focused on acute reperfusion therapies that are hampered by a very narrow therapeutic time window, it is essential to discover novel treatments that not only stop the progression of the ischemic cascade during the acute phase, but also improve the recovery of stroke patients during the sub-acute or chronic phase. In this regard, several studies have shown that endothelial progenitor cells (EPCs) can repair damaged vessels as well as generate new ones following cerebrovascular damage. EPCs are circulating cells with characteristics of both endothelial cells and adult stem cells presenting the ability to differentiate into mature endothelial cells and self-renew, respectively. Moreover, EPCs have the advantage of being already present in healthy conditions as circulating cells that participate in the maintenance of the endothelium in a direct and paracrine way. In this scenario, EPCs appear as a promising target to tackle stroke by self-promoting re-endothelization, angiogenesis and vasculogenesis. Based on clinical data showing a better neurological and functional outcome in ischemic stroke patients with higher levels of circulating EPCs, novel and promising therapeutic approaches would be pharmacological treatment promoting EPCs-generation as well as EPCs-based therapies. Here, we will review the latest advances in preclinical as well as clinical research on EPCs application following stroke, not only as a single treatment but also in combination with new therapeutic approaches.

## Introduction

Ischemic stroke remains as one of the leading causes of mortality and disability in Europe (1, 2). Following the ischemic insult, two large areas can be usually distinguished: the ischemic core, infarcted tissue with irreparable damage; and the penumbra area, surrounding the ischemic core, which contains hypoperfused tissue that is still viable (3). Following the occlusion of the blood vessel, the pathological paths of the ischemic cascade are activated, which eventually leads to neuronal death by either necrosis or apoptosis (4, 5). Hypoxic environment triggers glutamate excitotoxicity through a massive release of glutamate after injury, which provokes neurotoxicity by binding to N-methyl-D-aspartate receptors (NMDAR), promoting the entry of large amounts of Ca^2+^ in neurons (4–8). In addition, high concentrations of intracellular Ca^2+^ produce neuronal damage by increasing endoplasmic reticulum (ER) stress, reactive oxygen species (ROS), and depletion of ATP-depending processes (4, 5). Moreover, all together lead to the uncoupling of the mitochondrial electron chain, further increasing ROS and cellular death (4, 5). Overall, it is estimated that 2 million neurons die for every minute of obstruction (9).

Among others, mechanical thrombectomy and thrombolysis by the recombinant tissue plasminogen activator (rtPA) are effective therapeutic approaches by restoring the blood flow (10–17). However, these reperfusion therapies are hampered by a very narrow therapeutic time window, therefore it is necessary to search for alternatives and/or complementary treatments encompassed in, monitoring of cerebral homeostasis, reperfusion, neuroprotection and neurorepair strategies (12, 18, 19). In fact, angiogenesis and vasculogenesis represent two targets to obtain improvements in the outcome of patients following ischemic stroke (20–22). Whereas, angiogenesis means the sprouting of new blood vessels from uninjured ones, vasculogenesis is the *de novo* formation of blood vessels driven by endothelial progenitor cells (EPCs) (23). Importantly, it has been suggested that proinflammatory environments (e.g., following stroke) may enhance the proliferation and angiogenic function of EPCs (24). Therefore, both endothelial cell repair-regeneration and tissue neovascularization appear as key processes to restore the blood supply in the cortical areas affected by the vessel occlusion.

EPCs are circulating cells that exhibit both, progenitor- and stem-cell characteristics, since they have the capacity to differentiate into mature endothelial cells and self-renewing (25, 26). In the late 90's, Asahara et al. were the pioneers to identify EPCs in peripheral blood (27), and to determine their medullar origin (20). However, there is controversy nowadays regarding the origin of EPCs as both vascular and white adipocytic niches have been suggested over the years (28–31). The main function of EPCs is the maintenance of the endothelium by either releasing angiogenic growth factors or acting as a cellular reservoir for the replacement of injured endothelial cells (25, 32, 33). It is widely established that EPCs can be defined according to their angiogenic properties: early-outgrowth EPCs and late-outgrowth EPCs (25, 32, 33). On one hand, the early-outgrowth EPCs, or colony-forming unit endothelial cells (CFU-ECs), participate in the maintenance of the endothelium by the paracrine release of different pro-angiogenic factors (33). On the other hand, late-outgrowth EPCs, or endothelial colony-forming cells (ECFC), are characterized by their capacity to differentiate into mature endothelial cells, being the main player in the *de novo* formation of blood vessels (33). Late EPCs can also secrete several angiogenic factors, but in smaller quantities than early EPCs (34). Furthermore, these two types of EPCs can also be differentiated/classified by their *in vitro* characteristics: whereas early EPCs appear within a few days of culture and form colonies with spindle-shaped cells surrounding them, late EPCs appear at 2–3 weeks and show a cobblestone shape (34). Finally, kinase insert domain receptor (KDR) (also known as vascular endothelial growth factor receptor 2, VEGFR-2), CD34, and CD133 are the most common surface markers to detect EPCs (25). However, there are discrepancies regarding CD133 since it was also established as a specific marker for early EPCs (33). In addition, CD146 and von Willebrand factor (vWF) are characteristic markers of late EPCs (33). Overall, unfortunately, a unique marker to define EPCs clearly is still lacking.

**Figure 1:**
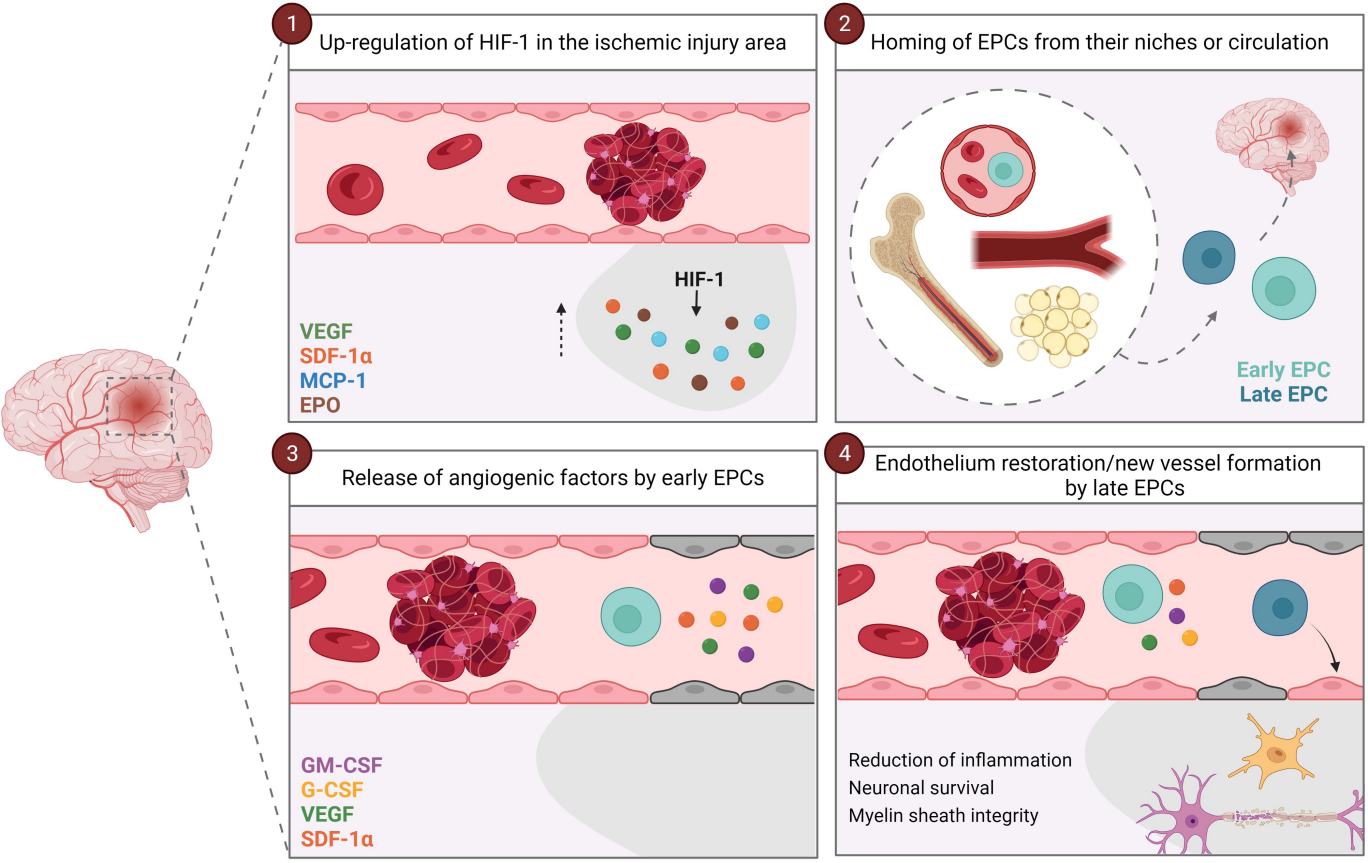
Vascular repair mediated by EPCs. After an ischemic stroke, HIF-1 is up-regulated and induces the expression of VEGF, SDF-1α, MCP-1 and EPO in the ischemic injury area **(1)**. These factors induce the homing of EPCs from their niches (bone marrow, vascular niches or white adipose tissue) or circulation to the ischemic brain area **(2)**. Firstly, early EPCs arrives at the site of injury, and paracrinally release factors that promote angiogenesis and recruit late EPCs **(3)**. Once the late EPCs arrive at the ischemic lesion zone, they restore the endothelium or from new vessels guided by the early EPCs **(4)**. In addition, both early and late EPCs reduce inflammation and promote neuronal survival and myelin sheath integrity. EPCs, endothelial progenitor cells; EPO, erythropoietin; G-CSF, granulocyte-colony stimulating factor; GM-CSF, granulocyte macrophage-colony stimulating factor; HIF-1, hypoxia-inducible factor-1; MCP-1, monocyte chemoattractant protein-1; SDF-1α, stromal-derived factor-1α; VEGF, vascular endothelial growth factor.

Several studies have demonstrated that different factors mobilize EPCs from their niches, such as granulocyte macrophage-colony stimulating factor (GM-CSF) (35, 36), granulocyte-colony stimulating factor (G-CSF) (37, 38); vascular endothelial growth factor (VEGF) (39–42); stromal-derived factor-1α (SDF-1α) (41–44); and erythropoietin (EPO) (45). Interestingly, some of these factors (e.g., VEGF, GM-CSF, and G-CSF) are also released by EPCs as paracrine signaling, promoting angiogenesis and triggering the proliferation and migration of endothelial cells (46–48). Moreover, EPCs release different vasodilators and vasoconstrictors factors, such as nitric oxide (NO) through the endothelial nitric oxide synthase (eNOS) activity (34, 49), and the tPA (49); as well as anti-inflammatory molecules (50). In the recent years, the role of extracellular vesicles and exosomes has been described in cell-to-cell signaling (51–53). Interestingly, EPCs also release extracellular vesicles that participate in angiogenesis (53, 54). Therefore, all these features make EPCs an interesting target following cerebral ischemia as they modulate vasculogenesis and angiogenesis, either by stimulating re-endothelialization of injured vessels or by stimulating the formation of new ones (26, 55, 56). Specifically, hypoxia-inducible factor-1 (HIF-1) is up-regulated under hypoxic conditions, which activates the expression of VEGF and SDF-1α, among others; eventually inducing the homing of EPCs to the hypoxic tissues (44, 48, 55–58) (Figure 1). In addition to modulating vasculogenesis and angiogenesis, EPCs release different factors that reduce inflammation, promote neuronal survival, and maintain the myelin sheath integrity in the damaged tissue, the latter probably by regulating the survival and migration of oligodendrocytes through SDF-1α interaction (59, 60). In summary, early and late EPCs work synergistically for proper angiogenesis and revascularization: the high concentration of angiogenic factors and inflammatory cytokines in the ischemic injury area attracts early EPCs, then, early EPCs release different factors in a paracrine way that promote angiogenesis and recruit late EPCs to either restore the endothelium or form new vessels guided by the early EPCs (33) (Figure 1).

Therefore, EPCs appear as a promising target to find new therapies to overcome the ischemic damage, all thanks to their ability to both directly participate in blood vessel repair and remodeling, and indirectly release different trophic factors to stimulate the repairing processes following ischemic injury. Here, we will review the latest advances in preclinical as well as clinical research at EPCs application following stroke, not only as a single treatment but also in combination with new approaches.

## Relationship between EPCs levels and the prognosis following stroke

For years, the quantification of EPCs has been proposed as a possible surrogate biomarker of cardiovascular function, since low circulating levels of EPCs were associated with increased cardiovascular risk (61–63). Indeed, although EPCs have been related to endothelial cell regeneration and neovascularization after tissue ischemia (61, 64–66), such cerebrovascular risk factors reduce the baseline levels of circulating EPCs (67). Accordingly, stroke patients displayed a lower number of EPCs at the ischemic event onset than their healthy counterparts (65); and such a reduced level of EPCs was negatively correlated with systolic blood pressure, diastolic blood pressure, triglycerides, LDL, and fasting blood glucose, all considered as vascular risk factors (67). Interestingly, proteomic analysis of samples from patients with ischemic stroke suggested that their EPCs were in a more advanced differentiation state and had a lower capacity for proliferation than those from healthy subjects (68). This study reveals that cerebral ischemia not only acts locally but also at systemic level, and such a general impact may be related to the functional impairment, since EPC-mediated protective vascular effects are mainly related to its proliferation ability. Clinical studies showed that the body counterattacks by increasing serum levels of pro-EPCs proliferation factors following ischemic stroke (69–72). Likewise, there is an increase in the levels of VEGF, SDF-1α, and active matrix metalloproteinase-9 (MMP-9) in the first 24 h after the onset, which are well-known molecules involved in EPC proliferation (73–75). Interestingly, the serum levels of these markers at 24 h correlated with the increment of EPCs during the first week (71, 76); although this raise can be negatively influenced by inflammatory factors such as leukocyte levels (76). Recently, high serum levels of VEGF at 3 months post-stroke have been associated with worse long-term (2-year) functional outcomes after injury (77), suggesting that long-term values of pro-EPCs proliferation factors might not be as beneficial as an acute increase.

Although controversial (78, 79), it has been widely observed that higher levels of circulating EPCs are related to a better prognosis following ischemic stroke (80–85). Indeed, our group was the first one to demonstrate the correlation between EPCs levels and both the injury progression and outcome following ischemic stroke (80). We observed that patients with higher EPCs numbers during the first week after ischemic stroke showed a reduction of the infarcted area and a neurological improvement at days 7 and 90; as well as a better outcome at 3 months compared to those with lower levels of EPCs (80). Importantly, patients with good outcome had higher numbers of EPCs than those with poor outcome on day 7 and at 3 months after ischemic stroke, but not at admission; and so, these results support the alleged beneficial role of EPCs mobilization following cerebral ischemia (80, 85). Curiously, it has been observed that the functional properties of EPCs to migrate and promote angiogenesis have more effect on both clinical outcome and *in vivo* angiogenesis than the absolute cell number (85–87); and that all EPC subsets would contribute similarly to this post-stroke angiogenesis (85). This can be observed in the positive association between functional properties of EPCs and increased flow diversion and cerebral blood flow following ischemic stroke (88). Nevertheless, the EPC-related cellular mechanisms underlying endogenous vascular repair, better neurological and functional outcome improvements and smaller growth of infarct volume are not fully understood. In the recent years, microRNAs (miRNAs) have been described to modulate the expression of several genes, gaining a special interest as therapeutic molecules (89). Accordingly, the profiling of miRNAs identified several ones potentially involved in the EPC-mediated angiogenesis and eventually good functional outcome following ischemic stroke (85). The analysis of CD34^+^ cells revealed that only 9 miRNAs were differentially expressed in the patients with a good outcome: the downregulated ones (hsa-miRNA-22-5p and has-miRNA-32-3p) induce cell senescence, and the upregulated ones (miRNA-27b24, miRNA-181d25, and miRNA-328) are involved in cell migration and angiogenesis (85). Interestingly, those mechanisms underlying the mobilization of CD34^+^ cells are also present following hemorrhagic stroke (90–92). Along these lines, a study using a mouse model of intracerebral hemorrhage (ICH) revealed that the single-nucleotide polymorphism (arginine-to-proline substitution) present at the codon 72 of the human Tp53 gene (a tumor suppressor gene) is directly related to endothelial cell survival, EPCs mobilization and neovascularization (93). Moreover, ICH patients carrying the arginine allele had a delayed neovascularization and impaired functional outcome compared to those patients with the proline allele (93). In summary, further studies are needed to decipher the mechanisms by which EPCs are associated with cellular, neurological, and functional improvements following ischemic stroke.

## Targeting EPCs as a promising treatment following stroke

The direct use of EPCs as a possible treatment for ischemic damage has been studied for years in different animal models, in which intravenously EPC-based cell therapy was the most used one (Table 1). In addition, recent treatments based on the secretome of EPCs and drugs that mobilize EPCs from their physiological niches have been also addressed (Table 1). Regarding clinical studies, the number of published results using the direct administration of EPCs in human is scarce, and most of the studies are based on pharmacological therapies increasing the mobilization of circulating EPCs (Table 1).

**Table 1:** Summary of relevant preclinical and clinical studies targeting EPCs following stroke.

**Cell-based therapies**			
**Preclinical studies**			
**References**	**Study**	**Model**	**Main outcomes**
Moubarik et al. (94)	Intravenously injection of EPCs from human cord blood 24 h after transient MCAO	Rat	- A reduction in the number of apoptotic cells and reactive astrogliosis, an increase in capillary density, and a stimulation of neurogenesis at the ischemic area. - Significant functional improvement at 7, 10, and 14 days after MCAO compared to controls.
Rosell et al. (95)	Intravenously injection of EPCs from mouse spleen 30 h after permanent MCAO	Mouse	- Significant increases in capillary density in the peri-infarct area, and in axonal rewiring. - Significant improvement in forelimb strength.
Garrigue et al. (96)	Intravenously injection of EPO-primed EPCs from human cord blood 1 day after transient MCAO	Rat	- The injection of EPO-primed EPCs increased their homing ability and the cerebral blood flow as well as reduced the BBB disruption and cellular apoptosis at the ischemic hemisphere on day 14 post-stroke.
Li et al. (59)	Intravenously injection of SDF-1α-transfected EPCs from human cord blood 1 week after permanent MCAO	Mouse	- Increase in blood vessel density and myelin sheath integrity, enhancement in neurogenesis, angiogenesis, as well as the proliferation and migration of EPCs. - Reduction in brain atrophy and improvement in the neurobehavioral function.
Hong et al. (97)	Intravenously injection of EPCs from mice 2 h after transient MCAO and for 7 days	Rat	- Increase in angiogenesis, and reduction in ischemic volume and gliosis at 14 days post-injury. - Increase in motor coordination at 7 and 14 days post-injury.
Wang et al. (98)	Intravenously injection of adiponectin-transfected EPCs from rat bone marrow 1 h after transient MCAO	Diabetic rat	- Decrease in the infarct area, as well as in cellular apoptosis in the peri-infarct area at 14 days post-injury. - Increase in angiogenesis in the peri-infarct area at 14 days post-injury. - Significant improvement in neurological function at 7 and 14 days post-stroke.
Kadir et al. (99)	Intravenously injection of EPCs from rat after MCAO	Rat	- Improvement in barrier protection at 3 days post-MCAO.
**Clinical studies**			
**References**	**Study**	**Main outcomes**	
Fang et al. (100)	Intravenously injection of autologous EPCs 1 month after acute ischemic infarction + 4 years follow up	- No significant differences in neurological or functional improvements, except for the Scandinavia Stroke Scale score at 3 months post-injection.	
**EPCs-Derived exosomes/secretome therapies**			
**Preclinical studies**			
**References**	**Study**	**Model**	**Main outcomes**
Rosell et al. (95)	Intravenously injection of cell-free conditioned media from mouse EPCs 30 h after permanent MCAO	Mouse	- Significant increase in the peri-infarct capillary density. - Significant improvement in forelimb strength.
Maki et al. (101)	Intravenously injection of cell-free conditioned media from mouse EPCs 24 h and 7 days after permanent bilateral common carotid artery stenosis	Mouse	- Increases in vascular density, myelin, and mature oligodendrocytes in white matter. - Improvement in the cognitive function at 28 days post-injury.
Wang et al. (102)	Intravenously injection of either regular exosomes or miR-126-enriched exosomes from mouse EPCs 2 h after permanent MCAO	Diabetic mouse	- The application of miR-126-enriched exosomes were more effective in decreasing infarct size and increasing cerebral blood flow and microvascular density in the peri-infarct area. - Likewise, animals treated with miR-126-enriched exosomes exhibited higher increases in angiogenesis and neurogenesis as well as neurological functional recovery.
**Pharmacological treatments targeting EPCs**			
**Preclinical studies**			
**References**	**Study**	**Model**	**Main outcomes**
Lee et al. (103)	G-CSF treatment given 2 or 24 h or 4 or 7 days after transient MCAO and maintained for 3 consecutive days.	Rat	- G-CSF treatment increased the cerebral vasculature and the proliferation of endothelial cells compared to the control group. - G-CSF treatment improved the behavioral recovery and reduced the infarct volume, the inflammatory infiltration, the BBB disruption, and the hemispheric atrophy compared to controls. - Specifically, G-CSF applications starting at 2 h, 1 or 4 days after ischemia resulted in a better functional recovery and a greater reduction in hemispheric atrophy than injection starting at day 7. Moreover, the G-CSF injection starting at 1 day induced larger endothelial proliferation compared with injection starting at 7 days.
Pellegrini et al. (104)	EPCs transplantation + EPO treatment given 1 or 2 or 3 days after transient MCAO and maintained for 3 consecutive days.	Rat	- The combination of EPCs + EPO treatment showed the best improvement in early and long-lasting neurological status. - EPCs + EPO also was the most effective approach to decrease apoptosis and to increase angiogenesis and neurogenesis in the ischemic area compared to controls and groups receiving EPCs or EPO alone.
Wang et al. (105)	Atorvastin or G-CSF or G-CSF+SDF-1 treatments given either pre- or post-transient MCAO	Rat	- The combination of G-CSF + SDF-1 showed the best results by improving neurological performance, reducing both cerebral infarction and blood-brain barrier disruption, and promoting greater angiogenesis in the ischemic brain.
Dong et al. (106)	8 weeks of piperlongmine treatment prior to permanent MCAO.	Mouse with hypercholesterolemia	- Enhancement in the angiogenic ability of EPCs. - Reduction in the infarct volume. - Improvement in the neurobehavioral outcome.
**Clinical studies**			
**References**	**Study**	**Main outcomes**	
Schäbitz et al. (107)	Intravenously administration of G-CSF treatment (30 or 90 or 135 or 180 μg) within 12 h after ischemia onset and for 3 days	- No significant differences in the clinical outcome. - A beneficial effect was found linked to dose-dependent only in patients with DWI lesions between 14 and 17 cm3.	
Alasheev et al. (108)	Subcutaneously administration of G-CSF treatment (10 mg) within 48 h after ischemia onset and for 5 days	- No significant difference in cerebral infarct volume between the experimental and control groups.	
Floel et al. (109)	Intravenously administration of G-CSF treatment (10 μg) at least 4 months after ischemia insult and for 10 days	- No significant effect of the treatment on the test of hand motor function.	
Sobrino et al. (110)	Orally administration of citicoline treatment (2,000 mg) within 24 h after stroke onset and for 6 weeks	- The administration of citicoline increased the concentration of EPCs, with better benefits when combined with rtPA. - There was a reduction of infarct growth as well as neurological and functional improvement at 3 months post-stroke.	
Ringelstein et al. (111)	Intravenously administration of G-CSF treatment (135 μg) within 9 h after ischemia onset and for 3 days	- G-CSF treatment did not show significant results in mRS and NIHH scores at day 90, neither in Barthel index and infarct size at day 30. - A trend was observed regarding reduced infarct growth in the G-CSF group.	
Mizuma et al. (112)	Intravenously administration of G-CSF treatment (150 or 300 μg) within 24 h after ischemia onset and for 5 days	- Clinical outcome scores did not show any significant difference at 3 months.	

### Cell-based therapies using EPCs

In different animal models of middle cerebral artery occlusion (MCAO), the intravenous injection of late EPCs reduced the infarct volume and the number of apoptotic cells, promoted angiogenesis, neurogenesis, axonal rewiring and BBB protection at the site of injury, as well as a functional and behavioral improvement after ischemic injury (94, 95, 97, 99). Remarkably, the time-point post-ischemia treatment apparently has no effect on the beneficial outcomes, since some studies treated animals at 24 h (94, 95), and others at 2 h and then for 7 days (97). Recently, an interesting approach has been developed, in which EPCs were virally transfected with different cytokines to improve their therapeutic properties (59, 98). For example, EPCs transfected with adiponectin (98) or SDF-1α (59) have several benefits such as promoting angiogenesis, neurogenesis, remyelination, as well as attracting EPCs and other neuronal and oligodendrocyte progenitors to the cerebral ischemic tissues. Accordingly, this resulted in a larger vascular density, better functional recovery, and smaller infarct volume and apoptosis ratio when compared to animals treated with wild-type EPCs (59, 98). It is important to note that such pro-regenerative outcomes were seen at different time-window applications, 1 h (98), and 1 week (59); in different MCAO models, transient (98), and permanent (59); and even in animals with diabetes, a known cerebrovascular risk factor (98). Beyond ischemic stroke, intravenous injection of EPCs at 6 h post-ICH promoted positive effects by reducing edema, apoptosis, and BBB permeability; and increasing the expression of tight junction proteins (113). Moreover, the levels of pro-inflammatory cytokines such as interferon-γ, IL-6, and tumor necrosis factor-alpha were reduced, whereas those of anti-inflammatory cytokines such as transforming growth factor-β1 and IL-10 were increased (113). Importantly, the magnetic vectorization of EPCs has been tested with satisfactory results (114); which encourages to keep researching new approaches that improve the cell delivery to the ischemic area.

Despite these promising results in animal models of stroke, the number of published results and clinical trials regarding direct application of EPCs in patients is almost absent. This is remarkable when we have in mind the fact that EPCs can home to the damaged BBB and promote its restoration, which would be extremely beneficial for patients with ischemic damage. The work from Fang et al. exposed the outcomes after the intravenously transplantation of EPCs (100). Such results came from a small clinical trial (EPCs group, *N* = 5; placebo group, *N* = 6; NCT01468064 in *ClinicalTrials.gov*) over 4 years, where authors observed a slight improvement in neurological and motor functions following the application of autologous EPCs (100). Although no significant differences were found in those outcomes compared to control subjects (possibly due to the small group of the follow-up), the trend was promising enough to further study both neurological and functional benefits of EPCs infusion in a larger cohort. Similarly, previous I and II trials showed encouraging results following autologous transplantation of CD34^+^ cells in stroke patients, either at the acute/subacute phase (115, 116), or chronic phase (117). It is important to remark that the administration of different subtypes of EPCs (late or early) and/or their origin (bone marrow, cord blood or spleen) might influence the outcome following application and, therefore, they are aspects to have in mind when analyzing the data. Overall, this reinforces the need of increasing the number of clinical trials addressing the beneficial outcomes of EPCs transplantation following stroke.

### Therapies based on EPCs-derived secretome/exosomes

Although promising, the use of cell therapies in stroke implies several risks such as emboli, immunological incompatibility, or infection (50). Therefore, new and safer research lines for the treatment of stroke are mandatory to avoid such issues. In this regard, the impressive work of Rosell et al. demonstrated for the first time that the infusion of cell-free conditioned medium (secretome) from late EPCs at 1 day post-ischemia was sufficient to increase capillary density in peri-infarct areas and eventually improve functional recovery in mice undergoing permanent MCAO (95). Subsequently, the therapeutic benefit of secretome was tested in a mouse model of prolonged cerebral hypoperfusion by bilateral stenosis of the common carotid artery (101). Although the secretome was administered at 24 h and 7 days after starting cerebral hypoperfusion, the results were similar to those obtained by Rosell et al.: increased vascular density, myelin, and mature oligodendrocytes in the white matter as well as improved cognitive function (101). More recently, the secretome of EPCs derived from stroke patients has proved to restore BBB function and promote angiogenesis in *in vitro* models of ischemia (118). Regarding neurogenesis, the interaction between neural stem cells (NSCs) and endothelial cells has been suggested for years (22, 95, 119–123). In this crosstalk, NSCs provide a vasculotrophic support for endothelial cells (ECs) under hypoxic conditions *via* the VEGF/HIF-1α axis (122), whereas either EPCs- or ECs-derived secretome protect NSCs against ischemic damage [(95, 123), respectively]. Interestingly, *in vitro* studies from Jing et al. revealed that the culture of NSCs with EPC-conditioned medium from hypoxic conditions increased their proliferation rate likely through VEGF secretion (124). Similarly, EPCs-derived secretome increases the proliferative rate of oligodendrocyte precursor cells (OPCs) and decreases their apoptosis, as well as enhances myelination under hypoxic environments either *in vitro* (101, 125) or *in vivo* (101). Several growth factors might be exerting the beneficial role mediated by the EPCs-derived secretome on oligodendrocyte remodeling following ischemic damage, such as C-X-C motif chemokine 12, VEGF and angiogenin, among others (101, 125, 126).

One of the components of the secretome of EPCs are exosomes, a type of extracellular vesicle that participates in cell-to-cell communication by releasing its contents, as mentioned above (51–53, 102). In the case of EPCs-derived exosomes, they are known to participate in angiogenesis and even protect the endothelium from suffering further damage or dysfunction under the hypoxia-reoxygenation process (53, 54, 127). Notably, EPCs-exosomes reduced ischemic damage and apoptosis, preserved cerebral blood flow and vascular microdensity, promoted angiogenesis and neurogenesis, as well as, improved neurological function in a mouse model of permanent MCAO (102). Interestingly, this beneficial effect was enhanced by using exosomes enriched in miRNA-126, a molecule involved in vascular function and angiogenesis (102). In fact, one of the beneficial effects of practicing exercise before the stroke is that it increases miRNA-126 levels in EPCs-exosomes, which correlates with reduced infarct volume and apoptosis and increased microvascular density in mice undergoing permanent MCAO (128). Likewise, EPCs-exosomes transfected with miRNA-126 were uptaken by astrocytes enhancing their survival rate and reducing both cellular toxicity and ROS generation under *in vitro* hypoxia (129). Other miRNAs also have beneficial roles following ischemia, such as the miRNA-210 whose inclusion into EPCs-exosomes boosted their beneficial roles by improving the mitochondrial function in endothelial cells following hypoxia/reoxygenation damage (130).

As previously stated, the therapeutic application of EPCs still has some concerns, including the large number of cells needed for the treatment and their potential to become tumorigenic; and so, the attention is being focused on the use of EPCs-derived exosomes/secretome. Unfortunately, this field is in a preclinical stage, although the experiments with extracellular vesicles have demonstrated to exert similar beneficial roles to the cell-based treatment (118). Therefore, clinical trials and studies on humans are needed to confirm preclinical data regarding the use of EPCs-derived exosomes and/or secretomes as a more reliable and safer approach than cell-based therapies.

### Pharmacological treatments targeting EPCs

As mentioned previously, drug treatments to increase the number of EPCs appear as another interesting alternative to cell therapy. The application of GM-CSF, SDF-1α, statins, or EPO results in the mobilization of EPCs from their niches. Indeed, several works observed cellular and functional improvements following the administration of these drugs in rats undergoing either transitory (103, 104) or permanent MCAO (105). Overall, such pharmacological treatments resulted in a reduction of infarct volume, apoptosis, BBB disruption; an increase of angiogenesis and neurogenesis; an improvement of neurological function (103–105); and, interestingly, an increase in the levels of factors that induce homing of EPCs, such as VEGF or basic fibroblast growth factor (bFGF) in peripheral blood (105). Moreover, the levels of several growth factors involved in neurogenesis, such as glial cell-derived neurotrophic factor (GDNF), growth-associated protein-43 (GAP-43) and BDNF were also increased (104, 105). Importantly, there is a therapeutic synergy when combining GM-CSF with SDF-1α (105) or EPCs with EPO (104), which improves the effects of both drugs and cells. Notably, the injection of EPO-primed EPCs lead to an improvement in their migratory capacity, and it also had beneficial effects on the cerebral blood flow, apoptosis, and BBB disruption in a rat model of transient MCAO (96). Importantly, some of these drugs, and others, have been tested in patients where they showed therapeutic benefits. For example, patients treated with statins or citicoline (CDP-choline) after the stroke had a significant increase in the levels and mobilization of EPCs, which predicted with high sensitivity and specificity a good outcome at 3 months following injury (110, 131, 132). Likewise, the treatment with piperlongumine, an alkaloid extracted from *Piper longum*, exhibited anti-platelet aggregation and anti-inflammatory properties, and its administration significantly improved the functions of EPCs as well as reduced ischemic damage in an ischemic brain model of high-fat diet-fed mice (106). Although all these drugs promote the mobilization of EPCs, none is EPCs-specific as they induce homing of other progenitor cells and other beneficial effects. Therefore, cellular and functional improvements seen in patients with stroke treated with those drugs cannot be attributed exclusively to their effect on EPCs. The development of new drugs targeting exclusively EPCs is mandatory to confirm that the EPC-mediated beneficial effects are enough to promote functional recovery following ischemia.

Recently, the potential clinical use of the G-CSF is getting attention as it possesses a more specific activity spectrum (133). The G-CSF is a glycoprotein released by endothelium and immune cells that acts as a hematopoietic growth factor (134). Among other beneficial mechanisms following vascular injury, the G-CSF can promote angiogenesis by mobilizing EPCs from the bone marrow to the peripheral blood (135). This makes it a promising target to enhance vascular repair in CNS injuries. However, results in ischemic stroke patients could not find reliable improvements neither the clinical outcome nor infarct volume, even after a phase IIb trial (107–109, 111, 112). Noteworthy, a meta-analysis study did show a motor improvement, which was probably due to the increase in CD34^+^ counts, therefore, a higher mobilization of EPCs to the damaged area (136). Interestingly, there is an ongoing phase 1/2a clinical trial to evaluate the safety and efficacy of combining EPO and G-CSF treatments in patients with neurological diseases, including ischemic and hemorrhagic stroke (NCT02018406, *ClinicalTrials.gov*). Overall, further studies are mandatory to confirm the potential use of G-CSF as another alternative treatment following cerebral ischemia.

## Concluding remarks

As shown here, there is compelling evidence that support the potential of EPCs as a therapeutic target. Data from both preclinical and clinical studies showed promising results that are being slowly translated to clinical trials. Although the clinical trials have reduced the expectations regarding functional and cognitive outcomes; they do have shown the safety of the cell-based therapy (injection of EPCs) and drug treatments (G-CSF). Those clinical trials were performed in small cohorts of patients, and so, more trials with higher numbers are needed to confirm undoubtedly the impact of such therapies following cerebral ischemia. Finally, several preclinical studies have highlighted the beneficial role of EPCs secretome/exosomes, but no clinical trials using EPCs secretome have been carried out so far. Therefore, the use of such approach may overcome the concerning regarding EPCs cell-based therapies and provide a more specific spectrum than drug treatments.

## Author contributions

Conceptualization: AC, DR-S, and TS. Resources and supervision: LF, JC, and TS. Critical literature review: AC, MA-N, JMP-P, and DR-S. Writing—original draft preparation: AC, DR-S, JS-F, and TS. Writing—review and editing: AC, AO, MA-N, JS-F, PH, JP-P, AR, LF, JC, DR-S, and TS. Funding acquisition: DR-S, AC, LF, JC, and TS. All authors have read and agreed to the published version of the manuscript.

## Funding

This study was partially supported by grants from the Xunta de Galicia (PH, JC, and TS: IN607A2018/3, TS: IN607D 2020/09, AC: IN606A-2021/015), the Science Ministry of Spain (TS: RTI2018-102165-B-I00 and RTC2019-007373-1), and the Instituto de Salud Carlos III (AR: PI19/00186 and RD21/0006/0007). Furthermore, this study was also supported by grants from the INTERREG Atlantic Area (LF and TS: EAPA_791/2018_ NEUROATLANTIC Project), INTER-REG V A España Portugal (POCTEP) (LF and TS: 0624_2IQBIONEURO_6_E), the European Regional Development Fund (ERDF), the PT2020 program (LF: FEDER, project LABEL: POCI-01-0247-FEDER-049268), and the Fundação para a Ciência e Tecnologia (LF: project ENDEAVOUR: EXPL/BTM-ORG/1348/2021). Moreover, DR-S (CD21/00166), MA-N (IFI18/00008), and TS (CPII17/00027) are recipients of Sara Borrell, iPFIS, and Miguel Servet contracts, respectively, from the Instituto de Salud Carlos III. The funders had no role in the study design, data collection and analysis, decision to publish, or preparation of the manuscript.

## Conflict of interest

The authors declare that the research was conducted in the absence of any commercial or financial relationships that could be construed as a potential conflict of interest.

## Publisher's note

All claims expressed in this article are solely those of the authors and do not necessarily represent those of their affiliated organizations, or those of the publisher, the editors and the reviewers. Any product that may be evaluated in this article, or claim that may be made by its manufacturer, is not guaranteed or endorsed by the publisher.
